# Initial characterization of the large genome of the salamander *Ambystoma mexicanum* using shotgun and laser capture chromosome sequencing

**DOI:** 10.1038/srep16413

**Published:** 2015-11-10

**Authors:** Melissa C. Keinath, Vladimir A. Timoshevskiy, Nataliya Y. Timoshevskaya, Panagiotis A. Tsonis, S. Randal Voss, Jeramiah J. Smith

**Affiliations:** 1 Department of Biology, University of Kentucky, Lexington, KY, 40506, USA; 2 Department of Biology, University of Dayton, Dayton, Ohio, 45469-2320, USA; 3 Spinal Cord and Brain and Injury Research Center, University of Kentucky, Lexington, KY, 40506, USA

## Abstract

Vertebrates exhibit substantial diversity in genome size, and some of the largest genomes exist in species that uniquely inform diverse areas of basic and biomedical research. For example, the salamander *Ambystoma mexicanum* (the Mexican axolotl) is a model organism for studies of regeneration, development and genome evolution, yet its genome is ~10× larger than the human genome. As part of a hierarchical approach toward improving genome resources for the species, we generated 600 Gb of shotgun sequence data and developed methods for sequencing individual laser-captured chromosomes. Based on these data, we estimate that the *A. mexicanum* genome is ~32 Gb. Notably, as much as 19 Gb of the *A. mexicanum* genome can potentially be considered single copy, which presumably reflects the evolutionary diversification of mobile elements that accumulated during an ancient episode of genome expansion. Chromosome-targeted sequencing permitted the development of assemblies within the constraints of modern computational platforms, allowed us to place 2062 genes on the two smallest *A. mexicanum* chromosomes and resolves key events in the history of vertebrate genome evolution. Our analyses show that the capture and sequencing of individual chromosomes is likely to provide valuable information for the systematic sequencing, assembly and scaffolding of large genomes.

Vertebrate genomes encompass a broad range of genome sizes ranging from 340 Mb to ~130 Gb^1^. Notably, some of the largest and most complex genomes exist in species that are of critical importance to biomedicine and evolution. Tackling the challenge of assembling large vertebrate genomes will likely require new, hierarchical approaches for sequencing, assembly and scaffolding. Considerable progress has been made in recent years to enhance genomic and molecular resources for a species with a genome size that falls toward the upper end of the vertebrate range, the primary salamander model *Ambystoma mexicanum* (Mexican axolotl). For example, large-scale transcript sequencing efforts have enabled transcriptome, proteome, and quantitative trait locus analyses of vertebrate characteristics for which axolotls are particularly informative, including tissue regeneration, thyroid hormone signalling, paedomorphosis, and karyotype evolution^2^,^3^,^4^,^5^,^6^,^7^,^8^,^9^. Methods to create transgenic axolotls and manipulate gene functions are developing rapidly and are permitting in-depth functional analyses of known candidate genes^10^,^11^,^12^,^13^. Even with these advances, the axolotl lacks a fundamental resource to facilitate the use of modern, sequence-based methods of inquiry - a complete genome assembly.

The axolotl genome consists of 14 chromosome pairs (2N = 28)^14^ and estimates of its physical size range from 21–48 gigabases^15^,^16^,^17^. Early DNA reannealing studies suggested that repetitive elements constitute at least 70% of the *A. tigrinum* genome, a close relative of the axolotl; similarly large repetitive fractions are predicted for other salamander genomes^18^,^19^,^20^,^21^,^22^. Indeed, large genome size is a common feature of all extant salamanders, suggesting a shared period of genome expansion prior to the basal salamander divergence during the early Jurassic, ~180 million years ago^23^. Recent studies have documented expansions of intergenic and intronic regions in the axolotl genome, and increases in the lengths of these regions are associated with increases in the prevalence of potential functional elements and younger repetitive sequences^24^. Given our existing state of knowledge, it seems reasonable to conclude that axolotl genome structure was shaped by ancient expansions of mobile repetitive elements, and this created fertile landscapes for mobile element dynamics and DNA sequence evolution on more contemporary timescales.

Large genome size and repetitive DNA content are often cited as challenges for genome assembly, and the impact of these factors is dependent on the genomic distribution of repetitive and single copy sequences. If repetitive sequences are broadly interspersed throughout the genome, each copy (depending on length and identity with other copies) may be associated with a distinct break in assembly contiguity, potentially ranging from dozens to millions of breaks per repeat family. However, clustered repeats may be associated with a substantially smaller number of breaks. For example, centromeric repeats often represent a substantial fraction of a genome’s repetitive landscape but are localized to discreet genomic segments. In total, centromeric repeats are expected to break sequence contiguity at relatively few positions within a genome assembly. If the majority of repetitive sequences in salamander genomes are organized similarly, the development of a contiguous genome assembly may become a more tractable problem, with repetitive regions being localized to relatively few large assembly gaps. Thus far, little is known about large-scale distribution of repetitive elements in salamander genomes. A few large DNA fragments (~150 kb) for genic regions of the axolotl and newt genomes have been sequenced and assembled, indicating that the structure and distribution of repeats is compatible with genome assembly of sub-megabase intervals^24^,^25^. However, no salamander genome has been sampled sufficiently to establish the overall size and sequence composition of repetitive (and single copy) regions, and thus the inherent complexity of the genome assembly problem.

Here, we describe initial sequencing and analysis of the *A. mexicanum* genome. Over 6 billion shotgun sequence reads were generated to achieve 19× coverage of the genome. From these sequence data, we estimate the total size of the genome to be ~32 Gb, with the repetitive fraction representing approximately 40% of the total sequence length. Attempts to directly assemble these shotgun sequence data reveal the computational complexity of assembling the largest vertebrate genomes: assemblies fail due to memory limitations (beyond 1 terabyte of RAM). Given these computational constraints, we developed and implemented chromosome capture and sequencing approaches that permitted the assembly to be divided into tractable subsets, while also providing intrinsic scaffolding information. Sequence data from the two smallest axolotl chromosomes were used to: 1) validate and extend previous linkage mapping studies and 2) develop strategies for assembly and anchoring of individually sequenced chromosomes. Altogether these studies point to a multipronged approach that will permit the development of high quality genome assemblies for *A. mexicanum* and other salamander models.

## Results

**Figure 1:**
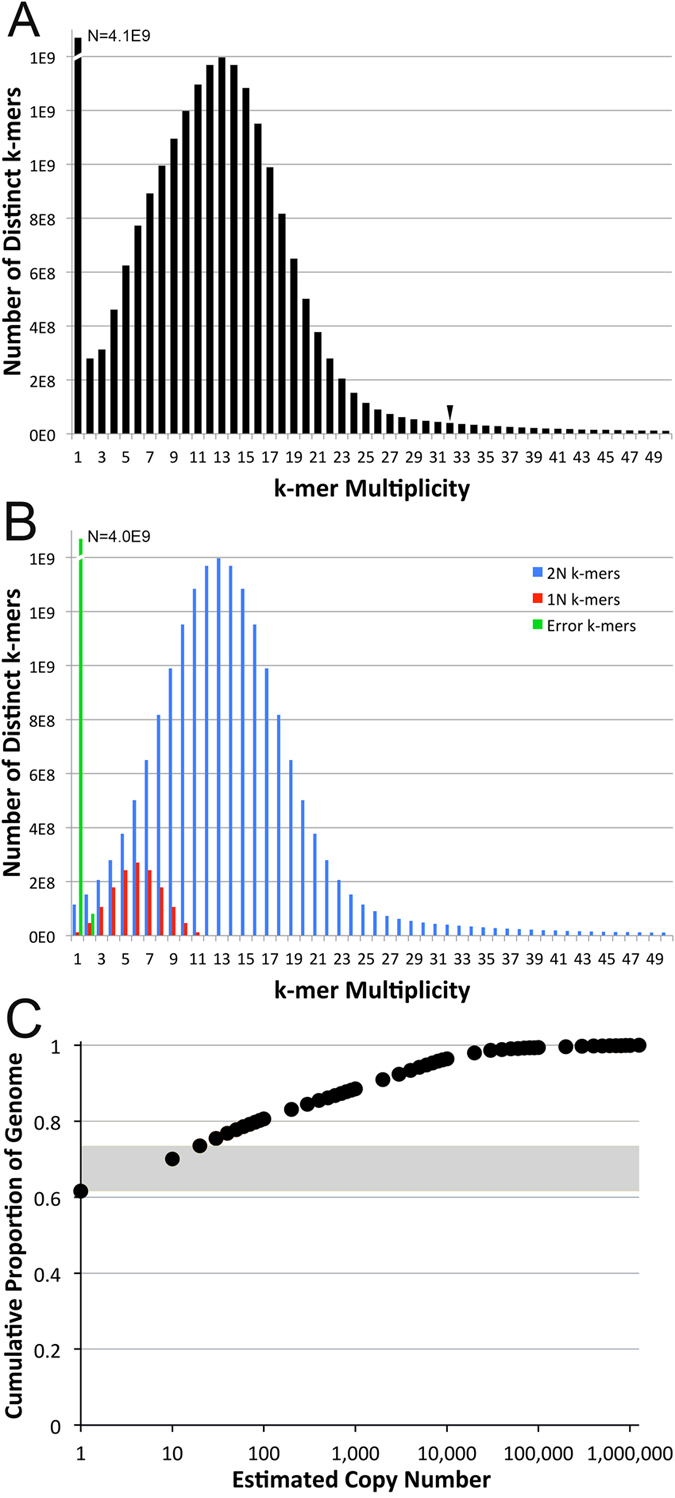
Distribution of 31-mer frequencies among >0.6 terabases of quality filtered sequence data generated from a single female *A. mexicanum*. (**A**) The observed distribution is humped with a peak at k-mer multiplicities of 13 and 14 (estimated mean of 13.50), presumably corresponding to k-mers that were sampled from the single-copy fraction of the genome. The k-mer multiplicity corresponding to 3 standard deviations above the mean of the single copy distribution (33.67) is marked by an arrow. (**B**) Decomposition of the observed distribution assuming symmetrical single-copy (diploid: 2N) and allelic (1N) k-mer distributions. The sum of all bins at a given multiplicity in panel B is equal to the observed multiplicity presented in Panel (**A**). (**C**) Low-copy k-mers account for the majority of *Ambystoma* shotgun sequence data and k-mers present at increasing copy number represent decreasing fractions of the shotgun dataset, suggesting that the diversity of repetitive sequences scales inversely with copy number. The region of the plot highlighted in grey represents copy number ranges that could plausibly exist at a copy number of ~1 per chromosome. The X-axis is plotted on a log scale to aid in visualization of patterns at lower estimated copy numbers.

A total of 16 lanes of Illumina shotgun sequence data (2 × 100 bp) were generated for a single female *A. mexicanum*, obtained from the standard laboratory strain maintained by the *Ambystoma* Genetic Stock Center (Animal #13003.1), yielding >0.6 Tb of raw sequence data. Analyses of k-mer frequencies were performed to estimate sequence coverage, genome size and repeat content. Filtered and quality trimmed sequences consisted of nearly 24 billion k-mers (at k = 31), with multiplicities ranging from one to 16 million. The k-mer sampling distribution contained a distinct peak at a coverage of ~13 (Fig. 1). Accounting for k-mer sampling across reads and ignoring presumptively erroneous k-mers, we estimated that this shotgun sequence dataset averaged ~19× sequence coverage across the genome. Correspondingly, we estimate the length of the *A. mexicanum* genome to be slightly over 32 Gb (32, 148, 237, 452 bp).

This shotgun sequence dataset was also used to generate an estimate of the relative size of the repetitive fraction of the axolotl genome, specifically as it relates to the assembly of sequences generated from short read chemistries. Based on k-mer sampling, we found that 60.1% of the genome (~19 Gb: modal coverage ± 3s.d.) can be considered effectively single copy, with the remaining repetitive fraction (13 Gb) occurring at copy numbers ranging from two to just over one million. In general the distribution of repetitive k-mers indicates that the diversity of repetitive sequences scales inversely with copy number, consistent with interpretations of previous DNA reannealing studies^18^,^22^ (Fig. 1C).

**Figure 2:**
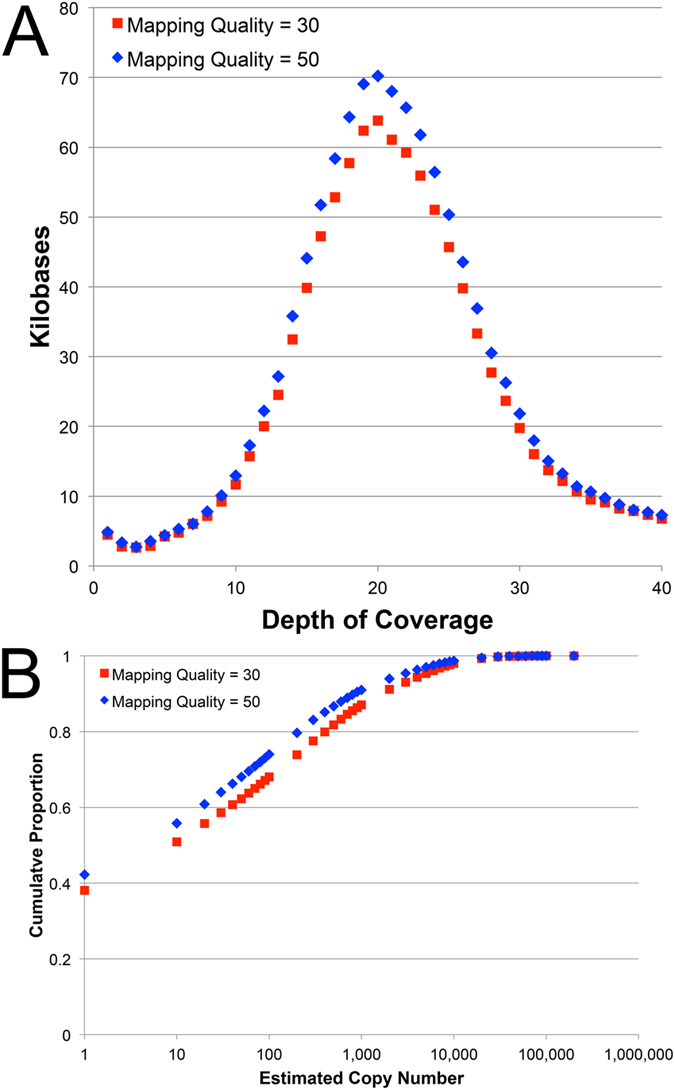
Estimation of sequence coverage and repeat content by alignment to assembled BAC clones. (**A**) The observed distributions are humped with peak depths of coverage between 19 and 20, consistent with estimates from analysis of k-mer frequencies. (**B**) Low-coverage bases account for ~40% of *Ambystoma* BAC sequence data and bases present at increasing copy numbers represent decreasing fractions of the BAC sequences, further suggesting that the diversity of repetitive sequences scales inversely with copy number.

To provide additional perspective on sequence coverage and repeat content, we also aligned our shotgun sequence dataset to a collection of 24 large genomic intervals that were assembled by BAC sequencing (covering 2.6 Mb, or just under 0.01% of the genome). These analyses corroborate our k-mer based coverage estimates and provide a similar perspective on the content of repetitive elements. In total ~40% of the assembled BAC sequence can be characterized as ~single copy (with depth of coverage ranging between 1 and 40×, Fig. 2). The remaining repetitive fraction shows a pattern consistent with k-mer based analyses but does not capture repeats at copy numbers in excess of 200,000. Extrapolating these estimates to the entire genome yields size estimates of ~13 Gb for the single copy fraction and ~19 Gb for the repetitive fraction. Presumably differences between alignment-based and k-mer based estimates reflect both increased sensitivity of alignment-based methods (which permit inexact matches) and the fraction of the genome considered.

**Figure 3:**
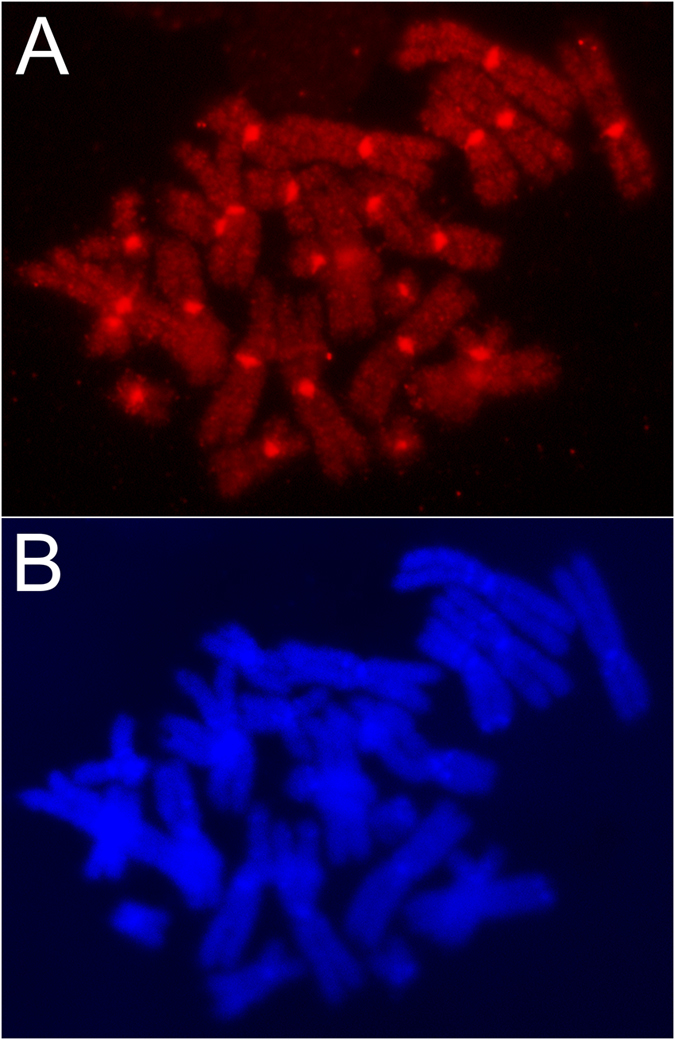
Distribution of repetitive elements in the axolotl genome. Chromosomes were hybridized with Cy3-dUTP labelled C_O_T DNA (Panel (**A**)) and stained with DAPI (Panel (**B**)). This fraction of C_O_T DNA contains the rapidly annealing (repetitive) portion of the genome and comprises ~45% of input DNA. Hybridization patterns show that repetitive DNA is heavily clustered at the centromeres and broadly distributed across all chromosomal arms.

While shotgun sequence data permit an assessment of the content of high-identity repetitive elements, they provide less information regarding the genome-wide distribution of high-identity repetitive elements or the abundance and distribution of transposable elements that have accumulated mutations over evolutionary time. To further assess the genome-wide distribution of high-identity repetitive elements, we performed *in situ* hybridization using the repetitive fraction of the genome (the most rapidly reannealing 40%, approximately corresponding to copy number >1 in Fig. 1C). Hybridization of sequences revealed that repetitive DNAs are both strongly localised to the centromeres and also interspersed across chromosomal arms at varying densities (Fig. 3). These analyses show that a relatively large fraction of repetitive DNA exists as interspersed elements. As discussed above, these interspersed repeats are expected to have the largest impact on assembly.

**Figure 4:**
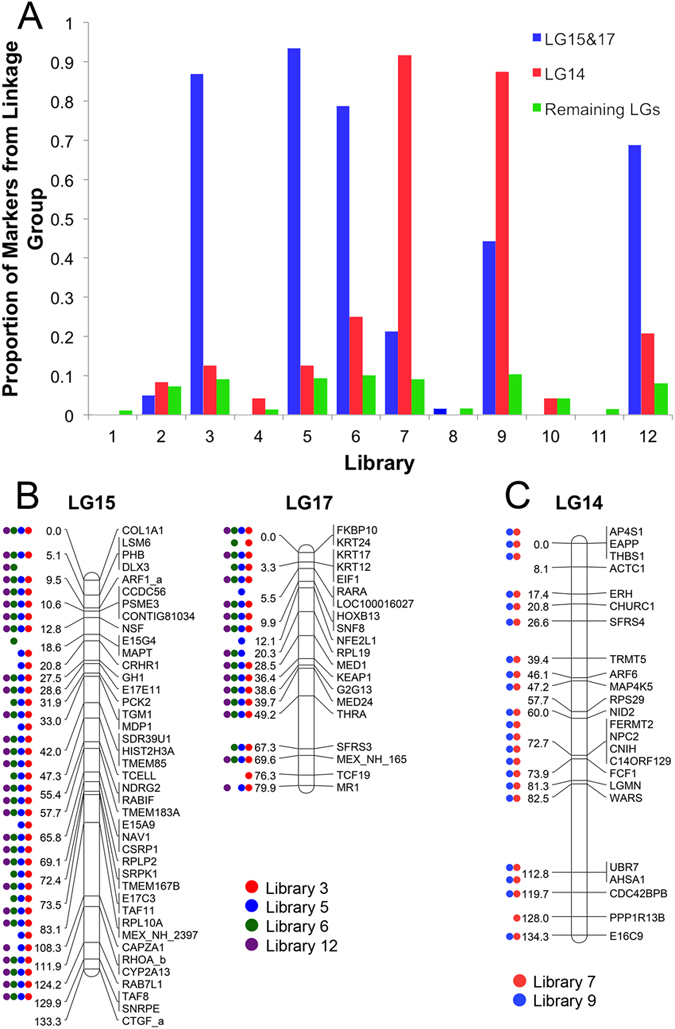
Mapping of reads generated by laser capture sequencing. Read mapping was used to assess the sensitivity and specificity of laser capture and amplification libraries. (**A**) The proportion of *Ambystoma* markers with nearly identical reads recovered from chromosome-targeted sequencing. Markers from target vs. off target linkage groups are presented separately. (**B**) The distribution of markers sampled from chromosome 13 (LGs 15 and 17) via targeted sequencing. Dots represent markers with mapped reads from each experimental series. Red, blue, green and purple dots denote markers that were sampled by reads (near perfect matches) from libraries 3, 5, 6 and 12, respectively. (**C**) The distribution of markers sampled from chromosome 14 (LG 14) via targeted sequencing. Red and blue dots denote markers that were sampled by reads from libraries 7 and 9, respectively.

In practice, the repeat content/distribution and size of the axolotl genome has presented major challenges to genome assembly. Attempts to directly assemble our shotgun sequence dataset failed due to memory limitations associated with traversing the de Bruijn graph structure during initial phases of contig extraction, despite the availability of one terabyte of RAM for these operations (Supplementary Text). In an attempt to circumvent these constraints, we turned our attention to developing a targeted sequencing approach that could rapidly generate data from smaller and discrete partitions of the genome while providing Gb-scale scaffolding/anchoring information. Using laser capture microscopy, individual mitotic dyads corresponding to the two smallest chromosomes of the axolotl karyotype were isolated for DNA amplification and sequencing. Notably, the axolotl karyotype is characterized by a graded series of chromosomal morphologies, which hampers the definitive identification of individual chromosomes. As such, we attempted to sequence individually amplified dyads in order to prevent cross contamination. In total, we generated and sequenced 12 barcoded libraries that are each derived from an individual dyad. Resulting reads were aligned to 918 transcribed sequences that were anchored to the *Ambystoma* genetic map. Initial analyses identified six libraries that were enriched for markers on specific *Ambystoma* linkage groups (Fig. 4A) and these were prioritized for further sequencing. Four of these libraries yielded nearly complete coverage of two separate LGs (LG 15 and 17; Fig. 4B). Replicated sampling of LG15 and LG17 markers among four independent libraries strongly suggests that these linkage groups comprise a single chromosome (AM13). Two additional libraries yielded almost complete coverage of a single linkage group (LG14; Fig. 4C), confirming that this small linkage group corresponds to a single chromosome (AM14). Overall, the fact that these libraries were heavily enriched in reads that map to the smallest *Ambystoma* linkage groups provided strong evidence that the sequencing approach was accurate and precise.

**Figure 5:**
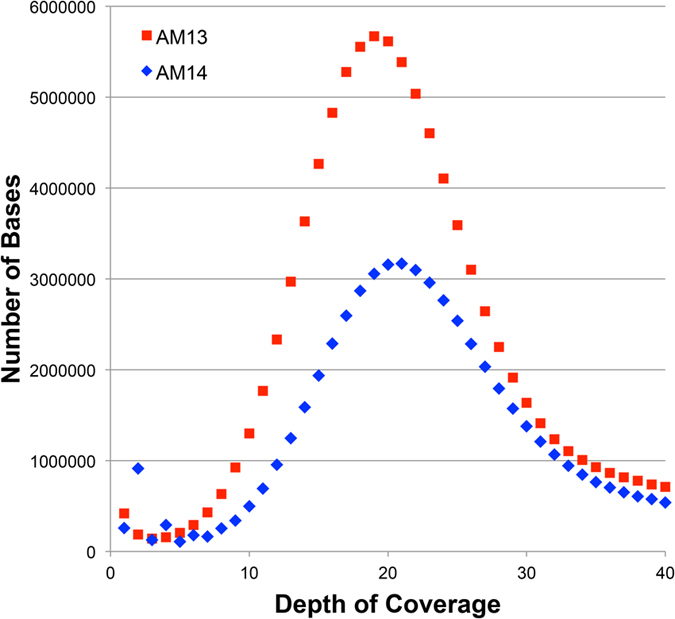
Estimation of coverage by alignment to assembled contigs from AM13 and AM14. The observed distributions are humped with peak depths of coverage between 19 and 20, consistent with estimates from alignments to BAC clones and analysis of k-mer frequencies. MQ30 = data are filtered to include only alignments with a map quality >= 30, MQ50 = data are filtered to include only alignments with a map quality >= 50.

To assess the utility of laser capture libraries in generating chromosomal assemblies, we performed several analyses using combined short-insert data from each of the two target chromosomes. In an attempt to correct for sequence errors associated with the preparation of amplified libraries, we also performed parallel assemblies that leveraged whole genome shotgun data to perform error correction and read filtering prior to assembly^26^. The relative decrease in the size and complexity of chromosome-targeted datasets (relative to the whole genome shotgun dataset) dramatically decreased the computational resources required for assembly. This allowed us to perform several rounds of assembly and optimize parameters for constructing and traversing de Bruijn graph structures. Each round of assembly required fewer than 6 hours to complete on a server with 512 GB RAM. The four optimized assemblies each exceeded 100 Mb in total length, with N50 scaffold lengths approaching 1 kb (Table 1). Error correction of amplified datasets decreased the number of singleton contigs, increased assembly N50 lengths and improved local scaffolding of both chromosomes. Alignment of chromosome-specific assemblies to our whole genome shotgun dataset also provided further corroboration of k-mer and BAC-based estimates of sequence coverage (Fig. 5).

**Figure 6:**
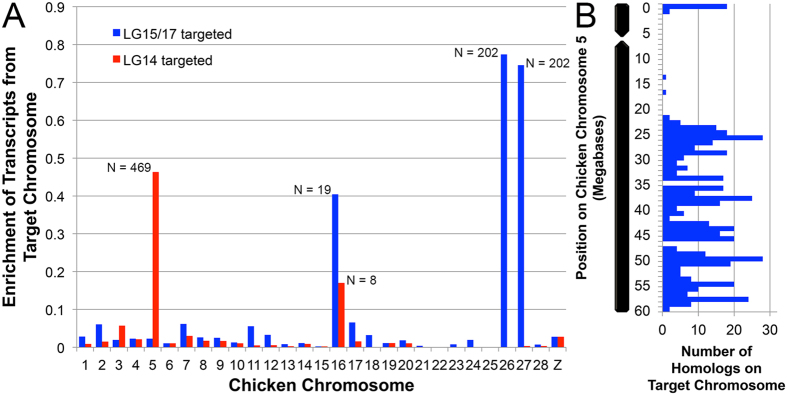
Conserved synteny between assembled *A. mexicanum* chromosomes and the chicken genome. (**A**) Tests for enrichment of AM13 (LG15/17 targeted) and AM14 (LG14 targeted) presumptive gene orthologs across all assembled chicken chromosomes. “Enrichment” is defined as the observed number of orthologs divided by the total number of genes that have been annotated to the chromosome^55^. (**B**) The distribution of AM14 orthologs along chicken chromosome 5 reveals a discontinuous distribution consistent with the interpretation that chicken chromosome 5 was shaped by an ancestral fusion event, and a subsequent pericentric inversion.

To complement and evaluate out targeted assemblies, we aligned all reference *A. mexicanum* transcripts^27^ to our draft chromosome assemblies. In doing so, we were able to place a total of 1141 reference genes on AM13 and 921 on AM14 (Supplementary Table 1). To independently assess the validity of these annotations, *A. mexicanum* genes were aligned to the chicken genome. Previous analyses have revealed strong conservation of synteny between chicken and *A. mexicanum*, and we therefore expected that similar patterns of synteny should be apparent among the larger set of genes that were annotated to AM13 and 14^3^,^7^. These alignments confirmed that AM13 homologs were heavily enriched on chicken chromosomes GG26 and GG27 (Fig. 6A). Similarly, AM14 homologs were heavily enriched on GG5. Closer examination of the distribution of AM14 homologs across GG5 revealed that AM14 homologs are distributed across two discreet regions, suggesting that GG5 was shaped by an ancestral fusion event and a subsequent pericentric inversion (Fig. 6B), with the remainder of GG5 being orthologous to *Ambystoma* LG6^7^. Alternately, patterns of conserved synteny between AM14 and disjunct regions of GG5 might be explained by an ancient subtelomeric duplication (paralogs of spectrin beta chain occur in both subregions of the chicken genome) or possibly errors in genome assembly. Altogether, these analyses indicate that our chromosome-specific assemblies provide an accurate, though fragmentary, representation of *A. mexicanum* coding regions and their associated flanking sequences.

**Figure 7:**
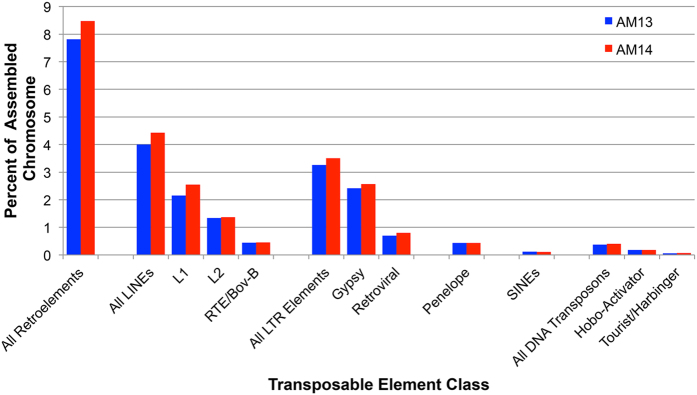
Summary of major repetitive element classes identified within assembled chromosomes. Percentages are shown separately for the two chromosomal assemblies. LINEs (Long Interspersed Nuclear Elements), LTRs (Long Terminal Repeat), Penelope and SINEs (Short Interspersed Nuclear Elements) are retroelement subclasses. Hobo-Activator and Tourist/Harbinger elements are DNA transposon subclasses. L1, L2 and RTE/Bov-B elements are LINE subclasses. Gypsy and Retroviral elements are LTR subclasses.

**Figure 8:**
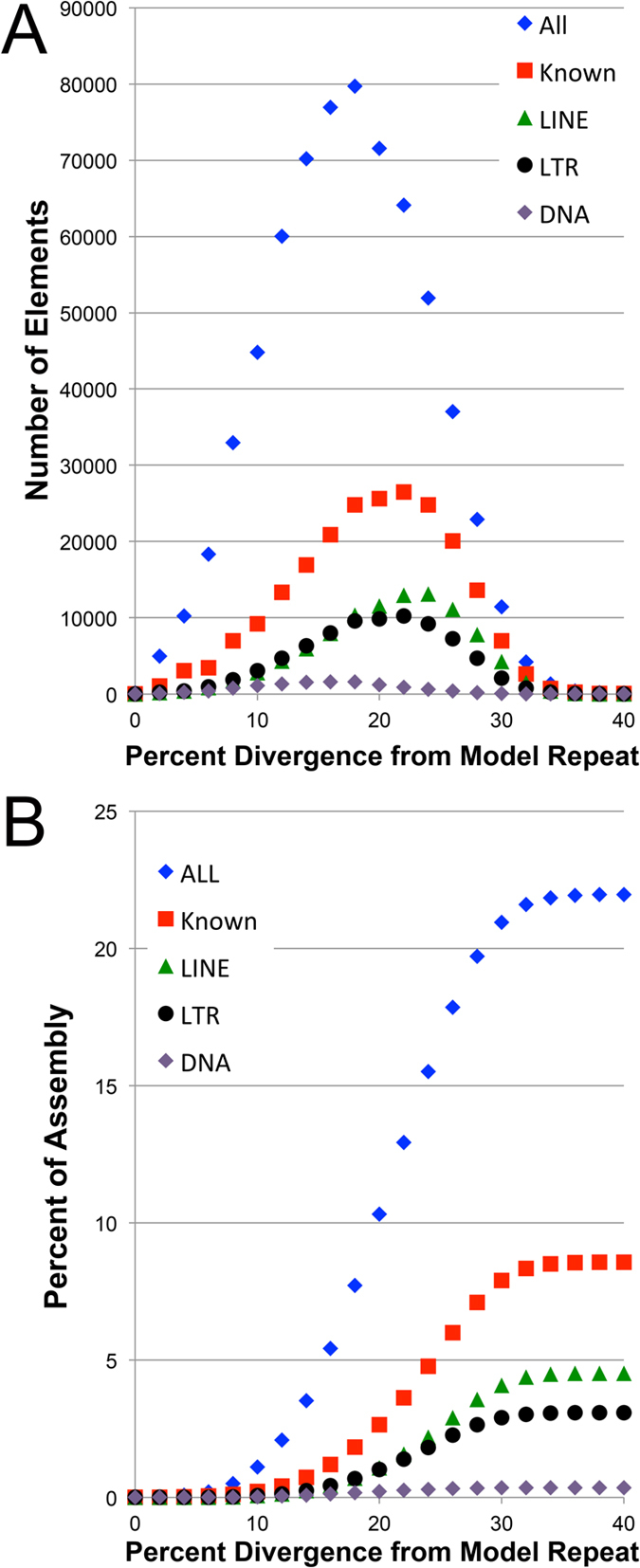
Diversity and abundance of repetitive elements in assembled scaffolds from AM13 and AM14. (**A**) Divergence between identified repeats and their RepeatMasker consensus sequence, using only information from *A. mexicanum* (model repeat). (**B**) The cumulative contribution (by length) of these same repeat classes. In both panels, patterns are shown for several classes. Known elements are comprised of LINEs, LTRs, DNA elements and other classes that are present at lower abundances (see Fig. 7). The class “All” consists of both known and unknown repeat classes.

Our chromosome-targeted assemblies also provided an opportunity to gain further insight into the abundance and distribution of divergent repetitive element copies that were active in the past but have subsequently accumulated mutations. These more divergent copies are more amenable to assembly and shed some light on the past activity of transposable elements and are expected to be underrepresented in the analyses described above. Repeat content was assessed using RepeatModeler/RepeatMasker, which classified 22% of assembled sequences as corresponding to identifiable repeat classes^28^,^29^. In total, 7% of the chromosomal assemblies could be assigned to known classes of repeats, although only 1.7% was assignable prior to de novo classification of salamander repetitive elements (Figs 7 and 8, Supplementary Table 2). Repeat counts for the separate chromosomal assemblies were remarkably similar and identified gypsy and LINE 1/2 elements as major contributors to the divergent repetitive fraction of the *A. mexicanum* genome (Fig. 7). Repetitive elements identified by this approach were typically divergent from their consensus sequence, with the typical element being ~20% divergent (Fig. 8). In total, 21.4% of the assemblable fraction could be attributed to a known or *de novo* identified repeat class.

## Discussion

Analyses of ~19× coverage shotgun sequence data from *A. mexicanum* provides an independent estimate of genome size that falls within the range of previous fluorometric estimates and further reveals that repetitive sequences are highly diverse in terms of sequence and copy number. In contrast to previous studies, we find that the majority of the genome consists of unique sequence^18^,^19^,^20^,^21^, at least with respect to assembly-relevant (short read) fragments. As might be anticipated, large genome size and complex repetitive environment present major challenges toward the development of a contiguous genome assembly for *A. mexicanum*. These initial studies indicate that chromosome-targeted sequencing presents an efficient strategy for simultaneously reducing assembly complexity and generating broad-scale scaffolding/anchoring information.

Our k-mer based analyses indicate as much as 62% of the genome (19.5 Gb) may be effectively single copy and that an additional 10–12% is potentially single-copy with respect to single chromosomes (i.e. at copy number less than ~20; Fig. 1C). Perhaps not surprisingly, alignment-based analyses yield smaller estimates for the single-copy fraction, yet at this scale ~12 Gb can be considered single copy (Fig. 2B). However, it is important to recognize that the designation “repetitive” is only relevant to its operational definition and that the designations “single-copy”, “low-copy” and “repetitive” are perhaps more relevant to the computational task of assembling genomes than they are predictive of the functionality of the underlying DNA segments^30^.

Regardless of the method used to identify repetitive sequences, it appears that high-identity repeats only partly account for the dramatic difference in genome size between salamanders and other tetrapod groups. The large size of the single/low-copy fraction is seemingly consistent with phylogenetic evidence suggesting that the axolotl’s large genome size traces its origins to an ancient expansion event. Several repetitive elements are identifiable in the assemblable fractions of AM13 and AM14, and on average these repetitive elements were 20% divergent from their consensus sequence. As has been observed for other salamander species, gypsy and LINE 1/2 comprise major fractions of identifiable repeat classes, suggesting that these elements have undergone active transposition in the relatively recent evolutionary history of several salamander lineages^31^,^32^, though notably LINE elements tended to be slightly more divergent than other repetitive element classes. With respect to large (and even human-sized) vertebrate genomes, it is likely that the vast majority of genomic DNA is derived from repetitive sequences^33^. The amplification of one or more repetitive elements almost certainly contributed significantly to the expansion of the ancestral salamander genome, although it may not be surprising that many of these sequences would have been heavily altered by mutations occurring over the last 180–200 million years. It seems plausible that this large volume of DNA may have provided raw material for the evolution of new functions in salamander genomes. Indeed, previous BAC sequencing studies have shown that salamander genes contain exceptionally long introns (which are transcribed into RNA) and that these introns contain a greater number and diversity of potentially functional secondary structures than their human counterparts^24^.

The preliminary assemblies presented here yielded hundreds of Mb of sequence data from two chromosomes that are scaffolded at an ~300 bp scale and anchored to individual chromosomes. We anticipate that laser-capture sequencing approaches will provide important information as computational resources and sequencing strategies continue to evolve. Chromosome-targeted sequencing approaches provide two major benefits with respect to the assembly of large genomes, namely large-scale anchoring and partitioning of the assembly into computationally tractable subsets. We anticipate that the generation of chromosome-targeted sequence data will become increasingly useful as a tool for subdividing genome assemblies, especially as it becomes possible to incorporate long read chemistries and associated algorithms into modern assembly pipelines^34^,^35^,^36^.

The results presented here also demonstrate the general feasibility of amplifying and deeply sequencing material from individual chromosomes that have been imaged and physically captured from the surface of a slide. Analyses of our shotgun datasets and draft assemblies illustrate the sensitivity and specificity of the approach and shed new light on the structure and gene content of the *A. mexicanum* genome. For example, these analyses confirm that *Ambystoma* LG14 corresponds to a single chromosome and improved the meiotic map by establishing that markers from LGs 15 and 17 should be coalesced into a single group, as previously proposed^7^. We anticipate that chromosome-specific libraries will continue to yield critical information for the hierarchical processes of scaffolding and assembling of the remainder of the *A. mexicanum* genome. We also anticipate that current assemblies will be immediately useful for studies that leverage *A. mexicanum* for basic and biomedical research, including the identification of proximate promoters and intron/exon boundaries, the development of molecular probes and the design of targeted mutagenesis constructs.

Based on previous cytogenetic observations, we anticipate that the smallest salamander chromosomes should be slightly larger than the largest human chromosomes, likely exceeding 250 Mb^37^,^38^, indicating that our laser capture/amplification approach will be applicable to a diversity of organisms, including those with genomes that are substantially smaller than that of *A. mexicanum*. Previous studies sequenced material from small numbers of pooled chromosomes that were captured from slides^39^ or within microfluidic devices^40^, which also provide invaluable scaffolding/anchoring information. The techniques employed here expand on these previous studies by circumventing the need to generate pooled samples, while generating deep sequence coverage of target molecules. Because our protocol uses commercially available reagents and reactions are performed at microliter scale, the approach should be feasible for any lab that has access to a laser capture microscope and standard laboratory equipment. The general approach outlined here can be readily adapted to a diversity of biological questions, including genomic characterization of microscopically identifiable cells (e.g. cancer or germ cells) or the development of chromosome-scale scaffolds for organisms that are not amenable to meiotic mapping or laboratory culture.

## Materials and Methods

### Ethics

All methods related to animal use were performed in accordance with AAALAC guidelines and regulations, under supervision of Division of Laboratory Animal Resources. Tissue collection was performed in accordance with protocol number 01087L2006, which was approved by the University of Kentucky Office of Research Integrity and Institutional Animal Care and Use Committee.

### Generation and analysis of shotgun sequence data

Library preparation and shotgun sequencing (Illumina HiSeq 2000) were outsourced to Hudson Alpha Institute for Biotechnology (Huntsville, Al). Resulting sequences were filtered to remove sequencing adapters using Trimmomatic^41^ and common contaminants (e.g. phiX) using Bowtie 2^42^. Initial k-mer analyses were performed using several values of k, as implemented by jellyfish^43^, and the final k-mer distribution (used to estimate genomic parameters and perform error correction) was calculated using Blue^26^. K-mer based estimates of sequence coverage, genome size and the size of the single copy fraction were generated using the method of Li *et al*^44^. K-mer based estimates of genome size assume a symmetrical sampling distribution for single copy regions and account for k-mer undersampling at the ends of short reads. Alignment-based analyses were performed by mapping individual WGS reads to assembled BAC sequences (GenBank accession numbers: 194293375–194293390, 325260854, 325260856, 325260858, 325260859, 325260861, 325260863, 325260865 and 325260867) using BWA mem (v.0.7.10) with default parameters^45^. Alignment files were filtered to remove unaligned reads using SAMtools (v.1.2)^46^, and both quality filtering and calculation of read depths were performed using sambamba (v0.5.4)^47^.

### Preparation of chromosomes

One hundred eggs from a wildtype axolotl cross were obtained from the *Ambystoma* Genetic Stock Center (AGSC) and maintained in an 18 °C incubator until they reached neurula stage (stage 17)^48^. Embryos were manually dechorionated with fine tip dissecting forceps and treated with 0.1% colchicine in 10% Holtfretter’s solution^49^ for 48 hours at 18 °C to promote the accumulation of metaphases. Colchicine-treated embryos were washed with fresh 10% Holtfretter’s solution then disaggregated using a Dounce homogenizer with loose pestle in a 0.075 M KCl solution (about 5 passes). After 45 minutes the swollen cells were fixed with 3:2 methanol:glacial acetic acid and stored in a −20 °C incubator. Fixed cells were spread on UV-treated membrane slides (0.17 mm PET Zeiss 415190-9071-000 for library “A” and 1.0 mm PEN Zeiss 415190-9041-000 for all other libraries) by pipetting 100 μl of fixed cell suspension directly onto the surface of the slide. Slides were pretreated by inversion over a steam bath for seven seconds immediately prior to cell spreading. Following spreading, slides were immediately placed in a steam chamber at approximately 35 °C for one minute, then dried by placing on a 60 °C hot plate for five minutes. The chromosomes were stained by immersion in a modified Giemsa stain (Sigma-Aldrich GS500-500 ML: 0.4% Giemsa, 0.7 g/L KH_2_PO_4_, 1.0 g/L Na_2_HPO_4_) for 2 minutes, rinsed in 95% ethanol, distilled water, and then air dried.

### Laser capture microdissection (LCM)

Single chromosomes were microdissected using a Zeiss PALM Laser Microbeam Microscope at 40× magnification. Microdissected chromosomes were pressure catapulted into clear adhesive cap tubes (Zeiss 415190-9191-000) and immediately processed through amplification.

### Preparation of amplified DNA

Dyads were released into solution by incubating overnight at 55 °C with 10 μl of a chromatin digestion buffer (1 mM EDTA, 20 mM TRIS pH 8.0, 0.2 mg/ml Proteinase K, 0.001% Triton X, in nuclease free water) added to the cap of the tube. Digested chromatin samples were briefly centrifuged and heat-treated for 10 minutes at 75 °C and 4 minutes at 95 °C to inactivate Proteinase K.

Chromosomal DNA was amplified using a Rubicon PicoPlex Whole Genome Amplification (WGA) kit (R30050) following standard manufacturer protocol, but substituting chromatin digestion buffer (above) for the cell extraction mix. The concentration and size distribution of amplified fragments were assayed using an Agilent 2100 Bioanalyzer (Agilent DNA 12000 Kit 5067–1508), and samples with less than 9 ng/μl were further amplified by performing two additional annealing/extension cycles. As an internal negative control, a piece of empty membrane was processed with each set of chromosome samples and run on the Bioanalyzer. Following initial quality control, 12 libraries were selected for outsourced sequencing on an Illumina HiSeq 2000 (Hudson Alpha Institute for Biotechnology, Huntsville, Al).

### Sequence analysis of amplified DNA

Leader sequences and common contaminants (e.g. phiX) were removed using Trimmomatic^41^ and the resulting reads were aligned to model transcripts of the *Ambystoma* linkage map^7^ or human reference genome, to identify nearly exact matches. To assess sequence coverage of the linkage map, chromosomal reads were mapped to model transcripts using the Burrows Wheeler Aligner (single-end mapping via the BWA-MEM algorithm)^50^. Reads were also aligned to the human genome using Bowtie 2 (paired-end mapping)^42^, in order to assess the degree to which off-target sequences might contribute to chromosomal fragment libraries. Concordantly mapping reads were considered potential contaminants.

Assemblies were generated using SOAPdenovo2 51. Assemblies of whole genome shotgun data were attempted using “pregraph” and “sparse pregraph” methods for constructing de Bruijn graphs, although neither approach yielded an assembly. Several iterations of chromosome specific assembly were performed, with the best assemblies employing error corrected data^26^ and broader coverage cutoffs to account for amplification bias during library construction (i.e. -c 0.05 -C 20)^51^. Alignments between *A. mexicanum* assemblies and reference transcripts were performed using megablast^52^ and alignments between *A. mexicanum* reference transcripts and the chicken genome were performed using blast (tblastx)^53^. Alignment-based estimates of genome coverage and repeat content were performed as described above.

### Preparation and labelling of C_O_T DNA

DNA was isolated using standard phenol/chloroform extraction^54^. DNA was adjusted to a concentration of 1,000 ng/μl in 1.2× SSC (1 ml), then sheared and denatured by heating to 120 °C for 2 min in a prewarmed aluminium block. Following denaturation, reannealing was performed by immediately placing the tube at 60 °C for 15 min and then immediately on ice. Remaining single stranded DNA was removed by adding S1 nuclease (Thermo Scientific #EN0321) to a final concentration of 100 U per 1 mg of DNA in 1× buffer, followed by incubation at 42 °C for 1 hr. C_O_T DNA was then purified via isopropanol precipitation^54^, reconstituted in TE buffer and labelled via degenerate oligonucleotide PCR. Briefly, 0.5 μg template DNA was amplified using the primer CCGACTCGAGNNNNNNATGTGG, GoTaq® DNA Polymerase and buffer, 200 μM d[A, C, G]TP, 100 μM dTTP and 100 μM Cy3-dUTP (Enzo) at a 25 μl reaction volume. Thermal cycling conditions were: 6 minute initial denaturation at 96 °C, 30 cycles of 94 °C for 1 minutes, 56 °C for 1.5 minutes and 72° for 2 minutes, followed by a final elongation 72 °C for 8 minutes.

### Classification of divergent repetitive elements

To characterize that divergent fraction of repetitive elements within the salamander genome (those not represented by high-count k-mers) we performed de novo searches for repetitive elements using RepeatModeler/RepeatMasker^28^,^29^. Repetitive elements were identified de novo using RepeatModeler and combined data from the AM13 and AM14 assemblies. Final repeat annotations were made using a combined dataset of elements from RepeatModeler and all known vertebrate repetitive elements contained in the RepBase 20.02 libraries. Estimates of sequence divergence were generated using RepeatMasker and a database consisting solely of repeats that were identified in the AM13 and AM14 assemblies.

## Additional Information

**Accession Codes**: Sequence data are deposited at the NCBI short read archives (http://www.ncbi.nlm.nih.gov/sra) under study number PRJNA269757. Assemblies are deposited at the NCBI GenBank (http://www.ncbi.nlm.nih.gov/genbank/) under accession number JXRH00000000.

**How to cite this article**: Keinath, M. C. *et al.* Initial characterization of the large genome of the salamander *Ambystoma mexicanum* using shotgun and laser capture chromosome sequencing. *Sci. Rep.* **5**, 16413; doi: 10.1038/srep16413 (2015).

## Supplementary Material

Supplementary Information

Supplementary Table 1

Supplementary Table 2

## Figures and Tables

**Table 1:** Summary statistics for de novo assembly of laser-capture sequence data from two chromosomes.

	Assembly			Contig		Scaffold	
	Length(Mb)	Number ofScaffolds	Number ofSingletons	N50 Length (bp)(Improvement)	ProportionScaffolded	N50 Length (bp)(Improvement)	Number>N50
LG15/17 (R)	302.5	604,617	243,354	231	0.598	705	136,682
LG15/17 (EC)	210.9	353,381	126,169	295 (28%)	0.643	830 (18%)	82,835
LG14 (R)	180.4	367,575	145,951	232	0.603	686	83,979
LG14 (EC)	143.0	258,214	93,931	290 (25%)	0.636	765 (12%)	62,022

Chromosomes correspond to *A. mexicanum* linkage groups 15/17 (LG15/17) and linkage group 14 (LG14). Statistics are presented for assemblies of raw sequence data (R) and assemblies of error corrected data (EC).
