# Supplementary material for: Initial characterization of the large genome of the salamander Ambystoma mexicanum using shotgun and laser capture chromosome sequencing
Source: Sci Rep. 2015 Nov 10;5:16413. doi: 10.1038/srep16413 (PMC4639759; doi:10.1038/srep16413)
Supplement: Supplementary Information [file srep16413-s1.pdf]

Supplementary Text

Initial characterization of the large genome of the salamander *Ambystoma mexicanum* using shotgun and laser capture chromosome sequencing.

Melissa C. Keinath, Vladimir A. Timoshevskiy, Nataliya Y. Timoshevskaya, Panagiotis A. Tsonis, S. Randal Voss and Jeramiah J. Smith

Behavior of genome assembly pipelines when processing paired-end whole genome shotgun reads.

At 32 Gb, the salamander genome is ~45% larger than the largest genomes assembled to date (e.g. the 22 Gb loblolly pine genome [1]). As such there are few experimental data that can be used to predict assembly performance for salamander. Toward this end, we have attempted to generate assemblies using several existing pipelines, including SOAPdenovo2 [2], SGA [3], ABySS [4], Fermi [5] and MaSuRCA [6], and are currently modifying these assemblers in an attempt to circumvent their limitations.

Assembly attempts with SOAPdenovo2 failed due to memory limitations (exceeding 1TB) during de Bruijn construction. We also attempted assembly using SOAPdenovo "sparse-pregraph" module in order to decrease memory overhead in graph construction. Initial traversal of the graph structure has not generated output after 30 days of compute time. By comparison, assembly of chromosome-specific libraries completed in less than 6 hours on a server with 512 GB RAM.

Assembly attempts with SGA require construction of a Ferragina-Manzini (FM) index of all reads [3]. As recommended by the developers, we generated the FM-index using the ropebwt algorithm [https://github.com/lh3/ropebwt]. The FM-index includes both a Burrows-Wheeler transform (BWT) of the data and a sample suffix array. The BWT was constructed in ~ 4 days using 200 GB of RAM. However generation of the SampledSuffixArray (*.sai file) failed, apparently due in part to internally encoded limits on the number of input reads.

Assembly attempts with ABySS were performed using a FM compressed representation of de Bruijn graph, called DBGFM [7]. The DBGFM was generated using KMC [8] to extract and count k-mers (k=31). A total of 896 million short unitigs were extracted using bcalm [7] and used to construct the DBGFM. Processing of the DBGFM was piloted using a single thread and >700 GB available RAM. This approach ran to completion using smaller datasets but failed to generate contigs for the *A. mexicanum* shotgun dataset, terminating with the error "misoriented vertex".

We also tested the package Fermi for assembly of low copy unitigs. After 19 days of run initial overlap graph was constructed and stored in gzip compressed MAG format as 219 GB file. Traversal of the graph by the "fermi clean" module terminated yielding no output or warning. Notably, termination of the run coincided with an attempt to allocate memory beyond 700 GB capacity of the machine in use.

Assembly attempts with MaSuRCA are ongoing and appear to be limited by both disk space and available memory. For loblolly pine, MaSuRCA successfully generated an initial fragment assembly of the 22 Gb genome using paired-end

illumina data generated from haploid megagametophyte tissue (completely lacking allelic variation that may confound assembly of super reads) [1]. Assembly of the loblolly pine genome completed in 3 months. Based on the developer's estimates, it is anticipated that assembly of the salamander genome via MaSuRCA will require in excess of 1 Tb of RAM, 10 Tb of disk space and 3 months of compute time.

References

1 Zimin, A. *et al.* Sequencing and assembly of the 22-gb loblolly pine genome. *Genetics* **196**, 875-890, doi:10.1534/genetics.113.159715 (2014).

2 Luo, R. *et al.* SOAPdenovo2: an empirically improved memory-efficient short-read de novo assembler. *GigaScience* **1**, 18, doi:10.1186/2047-217X-1-18 (2012).

3 Simpson, J. T. & Durbin, R. Efficient de novo assembly of large genomes using compressed data structures. *Genome Res* **22**, 549-556, doi:10.1101/gr.126953.111 (2012).

4 Simpson, J. T. *et al.* ABySS: a parallel assembler for short read sequence data. *Genome Res* **19**, 1117-1123, doi:10.1101/gr.089532.108 (2009).

5 Li, H. Exploring single-sample SNP and INDEL calling with whole-genome de novo assembly. *Bioinformatics* **28**, 1838-1844, doi:10.1093/bioinformatics/bts280 (2012).

6 Zimin, A. V. *et al.* The MaSuRCA genome assembler. *Bioinformatics* **29**, 2669-2677, doi:10.1093/bioinformatics/btt476 (2013).

7 Chikhi, R., Limasset, A., Jackman, S., Simpson, J. T. & Medvedev, P. On the representation of de Bruijn graphs. *J Comput Biol* **22**, 336-352, doi:10.1089/cmb.2014.0160 (2015).

8 Deorowicz, S., Kokot, M., Grabowski, S. & Debudaj-Grabysz, A. KMC 2: fast and resource-frugal k-mer counting. *Bioinformatics* **31**, 1569-1576, doi:10.1093/bioinformatics/btv022 (2015).
